# Psychometric properties of the German version of the Psychological Consequences of Screening Questionnaire (PCQ) for liver diseases

**DOI:** 10.3389/fpsyg.2022.956674

**Published:** 2022-08-11

**Authors:** Urs A. Fichtner, Andy Maun, Erik Farin-Glattacker

**Affiliations:** ^1^Institute of Medical Biometry and Statistics, Section of Health Care Research and Rehabilitation Research, Faculty of Medicine and Medical Center – University of Freiburg, Freiburg im Breisgau, Germany; ^2^Department for General Practice, Faculty of Medicine and Medical Center – University of Freiburg, Freiburg im Breisgau, Germany

**Keywords:** Liver screening, patient-reported outcomes, psychosocial consequences, validation, psychometrics, translation, screening consequences

## Abstract

**Background:**

This study aimed to translate the negative and positive items of the Psychological Consequences Questionnaire (PCQ) into German, to adapt this version to the context of screening for cirrhosis and fibrosis of the liver, and to test its psychometric properties.

**Materials and methods:**

The three subscales (physical, emotional, and social) were translated into German using a forward-backward translation method. Furthermore, we adapted the wording to the context of liver diseases. In sum, the PCQ comprises twelve negative items and ten positive items. We tested the acceptability, distribution properties, internal consistency, scale structure, and the convergent validity using an analysis sample of 443 patients who were screened for cirrhosis or fibrosis of the liver.

**Results:**

We found low non-response and non-unique answer rates on the PCQ items in general. However, positive items had higher non-response rates. All items showed strong floor effects. McDonald’s Omega was high for both the negative (ω = 0.95) and the positive PCQ scale (ω = 0.90), as well as for the total PCQ scale (ω = 0.86). Confirmatory factor analysis could reproduce the three dimensions that the PCQ intends to measure. However, it suggests not summing up a total PCQ score and instead treat the subscales separately considering a higher order overall construct. Convergent validity with the short form of the Spielberger State-Trait Anxiety Inventory (STAI-Y-6) was acceptable.

**Conclusion:**

Overall, our study results report a successful adaptation of the German PCQ with good performance in terms of acceptability, internal consistency, scale structure, and convergent validity. Floor-effects limit the content validity of the PCQ, which needs to be addressed in future research. However, the German version of the PCQ is a useful measurement for both negative and positive screening consequences - even in a non-cancer setting.

## Introduction

Medical screening is increasingly used in any medical discipline to detect the onset of diseases and prevent severe progression. Thus, screenings are commonly used in a population that has no acute symptoms. In Germany, a screening procedure called “Gesundheits-Check-Up” (health check-up) that aims to detect risk factors for certain diseases is available for statutory health insurance members aged 35 and higher and includes a blood and urine test (Institute for Quality and Efficiency in Health Care, 2022). A newly introduced screening procedure using a calculated ratio for the early detection of cirrhosis or fibrosis of the liver was tested in the context of the SEAL program (SEAL – Structured early detection of asymptomatic cirrhosis of the liver in Rhineland-Palatinate and Saarland) in two German Federal States (Rhineland-Palatinate and Saarland) from January 2017 to October 2021 (Nagel et al., 2019; Labenz et al., 2022). This program integrates the screening in the “Gesundheits-Check-Up” routine, which can be repeated every three years. The results of screening depend on cut-off values that have to be pre-defined. For the SEAL program, a cut-off value of the Aspartate aminotransferase to Platelet Ratio (APRI) of 0.5 was chosen. Thus, a positive screening rate of 3.5 to 4.0% is expected for the SEAL cohort with a false-positive screening rate of 70–80% (Unalp-Arida and Ruhl, 2017; Yip and Wong, 2017).

Beside the benefits of early detection of diseases, such as early treatment and potential prevention, negative effects should also be taken into consideration when evaluating the role of screenings (Harris, 2011; Institute for Quality and Efficiency in Health Care, 2022). In a review by Harris (2011), potential harms that cross multiple conditions, e.g., non-adherence, overdiagnosis and targeted screening are discussed. Landstra et al. (2013) contribute to this discussion by outlining that unrealistic optimism caused by negative screening results can lead to lower anxiety and thus to a reduction in health-promoting behaviors. A dilemma that occurs when introducing broad screening programs is that they tend to have low predictive power resulting in a high rate of false-positive results. Studies have shown that false-positive screening results have substantial negative psychosocial consequences (Cockburn et al., 1992; Ong et al., 1997; Brett et al., 1998; Brodersen et al., 2007). However, not only false-positive results can have that impact. Intensive surveillance during the screening process itself can produce unfavorable side effects on psychological well-being and health-related quality of life due to the confrontation with a potential threat (Rijnsburger et al., 2006). In order to identify potential burden in line with the screening, we conducted a cross-sectional survey alongside the SEAL study with screened participants.

Brodersen et al. (2007) emphasize the need for patient-reported outcome measures (PRO) with high content validity in order to systematically investigate psychosocial consequences of screening. To the best of our knowledge, no instrument exists so far to measure the psychological impact of screening for liver diseases, particularly not in German language. Therefore, we chose to adapt a questionnaire that was initially developed in the context of breast cancer screening. The Psychological Consequences Questionnaire (PCQ) was created 1992 in Australia and aims at measuring the positive and negative effects of breast cancer screening on emotional, physical and social functioning (Cockburn et al., 1992). To date, the PCQ was broadly used in measuring short-term screening impact in the context of mammography screening (Cockburn et al., 1992; Ong et al., 1997; Brett et al., 1998; Lowe et al., 1999; Bowland et al., 2003; Rijnsburger et al., 2006) and was also adapted to other types of cancer such as colorectal (Denters et al., 2013), anal (Tinmouth et al., 2011) and skin cancer (Risica et al., 2018). Cross-cultural adaptations produced translated versions of the PCQ in Dutch (Rijnsburger et al., 2006), Swedish (Olsson et al., 1999) and Danish (Brodersen and Thorsen, 2003) indicating a high usability of the instrument. We found no attempt for an adaptation of the PCQ beyond cancer diseases in the literature. However, we assume that liver cirrhosis is comparable to cancer in terms of the life-threatening perception of the population and thus consider the adaptation of the PCQ as suitable in this context. Consistent with the translation into Danish (Brodersen and Thorsen, 2003), we adapted not only the negative but also the positive items of the PCQ, but refrained from adding new items, as suggested by the authors in a later published work (Brodersen and Thorsen, 2008). The original PCQ covers three domains of normal functioning: physical, emotional and social functioning, both measured by negative and positive items. Emotional consequences are measured by five negative items (I3, I4, I5, I6, I12) and five positive items (I13, I14, I18, I19, I22). Four negative (I1, I2, I10, I11) and three positive items (I16, I17, I21) measure the physical aspects and three negative (I7, I8, I9) and two positive items (I15, I20) picture the social dimension (see Table 1). All items were rated on a 4-point scale ranging from 0 to 3. The exact scale labels differed slightly between negative and positive items and were 0 “not at all”, 1 “rarely” and “a little bit”, 2 “some of the time” and “quite a bit” and 3 “quite a lot of the time” and “a great deal.” They can be added to scores for each dimension after recoding the positive consequences to express the level of dysfunction.

**TABLE 1:** Distribution parameters: German version of the Psychological Consequences of Screening Questionnaire (PCQ) items.

Items			Non-response		Non-unique answers		Mean values		z-standardized Skewness	z-standardized Kurtosis	Distribution of extreme values	
								
			N	%	N	%	M	SD	Z-S	Z-K	%	%
											Undermost extreme	Upmost extreme
		
											(0 = not at all)	(3 = Quite a lot of the time/a great deal)

**Item No.**	**Subscale: Physical (P), Emotional (E), Social (S)**											
**Over the last week how often have you experienced the following things because of your thoughts and feelings about liver diseases? (negative items)**												
1	P	I had trouble sleeping	15	3.1	0	0	0.74	1.02	8.98	-1.32	59.5	8.9
		*Ich hatte Schwierigkeiten zu schlafen*										
2	P	I experienced a change in appetite	15	3.1	0	0	0.45	0.82	15.18	8.86	72.6	4.1
		*Ich hatte einen veränderten Appetit*										
9	P	I had difficulties doing things around the house which I normally do	15	3.1	1	0.2	0.60	0.93	10.92	1.08	66.4	5.2
		*Ich hatte Schwierigkeiten damit, Dinge zu Hause zu erledigen, die ich normalerweise erledige*										
10	P	I had difficulties meeting work or other commitments	15	3.1	0	0	0.58	0.91	11.55	2.41	65.7	5.2
		*Ich hatte Schwierigkeiten, meine Arbeit zu erledigen oder anderen Verpflichtungen nachzukommen*										
3	E	I have been unhappy or depressed	19	3.9	0	0	0.62	0.93	10.80	1.29	64.2	5.7
		*Ich war unglücklich oder niedergeschlagen*										
4	E	I have been scared or panicky	12	2.5	0	0	0.57	0.89	11.59	2.50	65.9	4.5
		*Ich war verängstigt oder beunruhigt*										
5	E	I felt nervous or strung up	13	2.7	0	0	0.65	0.94	10.46	1.07	61.3	6.3
		*Ich war nervös oder angespannt*										
6	E	I felt under strain	14	2.9	2	0.4	0.64	0.95	10.49	0.87	62.7	6.3
		*Ich habe mich belastet gefühlt*										
12	E	I felt worried about my future	12	2.5	1	0.2	0.90	1.11	6.80	-3.78	53.5	13.4
		*Ich habe mir Sorgen um die Zukunft gemacht*										
7	S	I found myself keeping things from those who are close to me	18	3.7	1	0.2	0.43	0.82	15.63	9.37	74.4	3.8
		*Ich habe Dinge vor mir nahestehenden Personen verheimlicht*										
8	S	I found myself taking things out on other people	16	2.3	1	0.2	0.41	0.78	16.15	10.99	74.5	3.2
		*Ich habe meine Laune an anderen Menschen ausgelassen*										
9	S	I found myself withdrawing from those who are close to me	16	3.3	1	0.2	0.46	0.86	15.31	8.76	73.0	5.2
d		*Ich habe mich von mir nahestehenden Personen zurückgezogen*										
**All things considered, would you say that your experiences in the SEAL study caused any of the following? (positive items)**												
16	P	I’m feeling more able to do things which I normally did before	41	8.4	3	0.6	0.94	1.02	4.96	-4.46	47.6	8.0
		*Ich fühle mich mehr dazu in der Lage, die Dinge zu unternehmen, die ich früher unternommen habe*										
17	P	I’m feeling more able to meet my home or work responsibilites	38	7.8	2	0.4	1.11	1.10	3.35	5.53	42.3	13.6
		*Ich fühle mich mehr dazu in der Lage, meinen Haushaöts- oder Arbeitsverpflichtungen nachzukommen*										
21	P	I can sleep better	38	7.8	0	0	1.04	1.08	4.49	-4.77	43.4	12.4
		*Ich kann besser schlafen*										
13	E	I feel assured that I do not have a liver damage	16	3.3	1	0.2	2.01	0.97	-5.97	-2.21	10.2	37.2
		*Ich fühle mich sicher, dass ich keinen Leberschaden habe*										
14	E	I feel more relaxed	25	5.1	0	0	1.67	0.95	-2.60	-3.48	14.3	19.9
		*Ich fühle mich entspannter*										
22	E	I have a greater sense of well-being all in all	33	6.8	0	0	1.35	1.06	0.81	-5.40	28.7	16.3
		*Ich fühle mich insgesamt wohler*										
18	E	I feel more hopeful about the future	33	6.8	0	0	1.42	1.08	0.04	-5.60	27.7	18.8
		*Ich blicke hoffnungsvoller in die Zukunft*										
19	E	I feel less anxious about liver diseases	28	5.7	2	0.4	1.40	1.03	0.72	-4.88	23.8	16.8
		*Ich habe weniger Angst vor Lebererkrankungen*										
15	S	My relationship with friends or relations improved	41	8.4	0	0	0.88	1.06	6.68	-3.38	52.0	10.6
		*Meine Beziehung zu meinen Freunden oder Angehörigen hat sich verbessert*										
20	S	I am getting on better with those around me	43	8.8	1	0.2	1.24	1.09	1.73	-5.68	35.2	14.8
		*Ich komme mit Menschen in meinem Umfeld besser klar*										

Items modified from Cockburn et al., 1992 under a CC BY license, printed with permission from Robert Sanson-Fisher original copyright 1992.

We found different reporting regarding the underlying factor structure of the PCQ. Olsson et al. (1999) and Rijnsburger et al. (2006) applied principal component analysis and identified three separate factors, one for each of the three dimensions. However, Rijnsburger et al. found only slightly higher item-own correlations of the PCQ subscales than item-other scale correlations indicating a certain overlap of the factors (Rijnsburger et al., 2006). Ong et al. (1997) also applied a factor analysis to examine the scale structure. Due to high cross correlations, they concluded that a one-factor-solution is more suitable for the PCQ. Those inconsistent findings motivated Cooper & Aucote to apply a confirmatory factor analysis (CFA) to identify the optimal factor structure. Their study found support for a one-factor solution.

So far, the majority of studies, including the most recent validation study of Cooper & Aucote, only focused on the negative PCQ scales (Cooper and Aucote, 2009). In our study, we aim to fill this gap by including also the positive subscale of the PCQ in the psychometric evaluation.

The aims of this study are as follows:

1. to translate the PCQ into German
2. to adapt it to the context of liver diseases
3. to assess acceptability, internal consistency, scale structure and convergent validity of the questionnaire in a screening population

By this, we aimed to produce an instrument that measures psychosocial consequences of screening in a liver disease context with acceptable psychometric properties.

## Materials and methods

### German version of the Psychological Consequences of Screening Questionnaire (PCQ)

In a first step, we slightly rephrased the items to fit to the context of liver screening. We then applied forward and backward translation procedures for all PCQ items. This method is demonstrated to be equivalent to the dual panel translation method for quality of life outcome instruments (Lee et al., 2019). First, two German native speakers with fluent command of English and social science background independently translated the PCQ into German and found consensus on their versions. Second, an English native speaker with fluent command of German and a bilingual professional translator translated the German text back into English. After minor revisions, the team found consent on a German version which was further discussed in a group of German native speaking Social Scientists (*n* = 5) with expertise in survey design. This expert assessment is strongly recommended to ensure high equivalence of the instrument (Lee et al., 2019).

### Study population and data collection

The SEAL program is a prospective study that aimed at evaluating a newly introduced medical screening method for early diagnosis of cirrhosis or fibrosis of the liver. From January 2018 to February 2021, patients who visited collaborating clinics or doctor’s offices in Rhineland-Palatinate or Saarland for a check-up were screened for liver cirrhosis and fibrosis. The screening process itself is a multistep design depending on the test result of each step (step 1: blood sample test and risk score, step 2: enhanced laboratory diagnostics and ultrasound, step 3: liver biopsy and enhanced diagnostics in a specialized clinic. Inclusion criteria for study participation were a minimum age of 35 and no known previous cirrhosis of liver (Nagel et al., 2019, 2020).

In August 2019, we contacted all patients who were included in the study so far. Due to ethical considerations, it was not possible to assess information on the phase in which the patients were in the whole screening process. This means that we could not gather information on whether patients with positive screening results already moved to step 2 or 3 or whether they still wait for an appointment. Therefore we could not control for false-positive results and potential effects caused by them. Since screening began in January 2018, it is plausible to assume, that the majority of the patients already received information on the test results. However, previous qualitative interviews revealed that patients, in general, receive no information in case of negative test results (results that showed no pathological findings) at all. A gross sample of 5.935 patients received a postal mail including a self-administered questionnaire, patient information and informed consent. With a return rate of 9% in those who were negatively screened and a return rate of 12% in those who were positively screened, we received 499 (negatively screened) respectively 21 (positively screened) completed questionnaires. In some cases, signed informed consent for our survey was missing. After a subsequent acquisition of missing consent documents, we excluded those cases that were not legitimate for evaluation (*n* = 34). We ended up with a net sample of *487 patient questionnaires* (*n* = 19 positively screened and *n* = 468 negatively screened). For this analysis, we included only cases with at least an 80% response rate to the PCQ items. This means we excluded cases that had more than three missing items on the negative PCQ scales and more than two missing responses on the positive PCQ scales. To increase comparability, we followed the approach of Rijnsburger et al. (2006) and imputed median scores per item in eligible questionnaires. Furthermore, in case of non-unique answers, we treated those as missing values. For psychometric analysis we ended up in an analysis sample of 443 cases. For detailed patient characteristics, see Supplementary File.

### Statistical analyses

For cross-cultural comparability, the analyses mainly followed the procedures of Rijnsburger et al. (2006). However, we slightly extended our psychometric test strategy according to Schupp et al. (2018) and Hayes and Coutts (2020) to reach a better understanding of the psychometric properties of the PCQ. Furthermore, we applied CFA using weighted least square mean and variance adjusted (WLSMV) estimators treating the four response categories as categorical. This method is more suitable in case of violation of normality assumption, which is the case here (Li, 2016). The analyses were conducted in IBM SPSS Statistics 26 and R using the lavaan package (Rosseel, 2012).

### Distribution properties

To give insights into the distribution properties, we computed skewness and kurtosis. Non-response rates and double cross rates (non-unique answers) for each item were shown to understand acceptability of the scale. Additionally, items with high skewness or kurtosis as well as items that show ceiling or floor effects were identified. We followed the classification for ceiling and floor effects as suggested by MacHorney & Tarlov, Varni et al. and Lin et al. which suggests a percentage of 0 to 15% as small, 16 to 30% as moderate and more than 30% as substantial floor or ceiling effect (MacHorney and Tarlov, 1995; Varni et al., 1999; Lin et al., 2013). Furthermore, we showed the 25th, 50th and 75th percentiles of the subscales and the total PCQ.

### Internal consistency

We computed McDonald’s Omega for the PCQ scales to evaluate internal consistency (Hayes and Coutts, 2020). Here, we deviate from the work of other earlier PCQ adaptations, where Cronbach’s Alpha was used. However, since there is a strong criticism about the use of alpha and since a congeneric model shows more realistic results, we decided to follow another approach (Dunn et al., 2014). We also assessed item-total correlation as well as mean-inter-item correlation for each subscale. Furthermore, we tested whether McDonald’s Omega allows computing an overall PCQ including both negative and (recoded) positive items.

### Scale structure

To confirm the prior findings in the literature, we both tested a three-factor solution, as well as an overall PCQ score as a single concept of adverse psychosocial consequences. Furthermore, we applied this testing rationale both for the negative and positive items. Since latter received little attention in the past, we also tested, whether an overall consideration of both the negative and positive PCQ items together is purposeful.

We conducted confirmatory factor analyses (CFA) to confirm the item-factor-relationship. Due to the controversial debate about the three- or one-factor-structure of the PCQ, we applied a four-step approach. First, we estimated a three-factor-solution not allowing covariance between the factors (strict model). Second, we modeled one-factor solutions. Third, we allowed covariances between the three dimensions and modeled a higher order factor model with three latent factors (physical, emotional, social) on level 1 and an overall PCQ factor on level 2. Fourth, we checked modification indices for plausibility and estimated a modified higher order model.

In detail, WLSMV estimator was used to compute robust standard errors and a mean- and variance-adjusted test-statistic. Model fit was evaluated using Chi^2^ goodness of fit test, robust Comparative Fit Index (CFI) (Hu and Bentler, 1999), robust Tucker-Lewis Index (TLI) (Tucker and Lewis, 1973), robust root mean square error of approximation (RMSEA) and robust standardized root mean square residual (SRMR) to determine local fit. A Chi^2^/df ratio below 3 indicates that the assumed model acceptably fits the data well (Moosbrugger and Kelava, 2020). We consider CFI and TLI values above 0.90 as an indication of a good fit (Moosbrugger and Kelava, 2020). RMSEA values below 0.1 are considered as moderate and values below 0.05 as good fit (Lin et al., 2013). SRMR values below 0.10 were considered as acceptable fit (Moosbrugger and Kelava, 2020).

### Convergent validity

To evaluate convergent validity, we correlated the PCQ subscales with the Spielberger State-Trait Anxiety Inventory (STAI Short version Y-6)^1^ (Marteau and Bekker, 1992). This scale is designed to measure current emotional status and is assumed to correlate high (Pearson’s r greater than 0.5) with all three subscales of the PCQ. We further hypothesize that the highest correlation to be found is with the emotional subscale of the PCQ. If this hypothesis cannot be rejected, we conclude acceptable convergent validity of the German version of the PCQ. Additionally, we assume that the STAI-Y-6 scale correlates stronger with the negative PCQ subscales than with the (recoded) positive PCQ subscales. This assumption is based on the rationale that the absence of a positive effect (e.g., not experiencing greater well-being) should not be considered as equal to the occurrence of a negative effect. This circumstance led Brodersen & Thorsen to rephrasing the positive items, so that they allow changes in both directions (Brodersen and Thorsen, 2003, 2008).

## Results

### Acceptability

We noticed low item-non-response for all PCQ items in our sample. Following the approach explained above, we had to exclude 15 cases for the negative PCQ items and 66 cases for the positive PCQ items since they show less than 80% of overall scale response. This pattern illustrates that the general non-response rate is higher for the positive PCQ items (ranging from 3.3 to 8.8%) than for the negative items (ranging from 2.3 to 3.9%) (see Table 1). We could not identify any non-response pattern that is associated with a specific subscale. Non-unique answers were generally low and were not observed more than three times per item (highest non-unique answer rate of 0.6% in item “feeling more able to do things which I normally did before”).

### Distribution properties

For all negative PCQ items we found mean values ranging between the categories “0 Not at all” and “1 Rarely” indicating that the impact of screening is generally low. This pattern also explains a substantial floor effect for all negative items with more than 50% of responses lying in the undermost category. In contrast, we found small ceiling effects with the highest percentage of values in the item “I felt worried about my future”. As consequence, the data is highly skewed. Z-standardized skewness exceeds the cut-off value of + 1.95 for any negative PCQ item. Regarding kurtosis, items 2, 4, 6, 9, 10, 11, and 12 lie beyond the range of −1.95 to + 1.95 indicating no normal distribution.

The positive PCQ items generally show higher mean values ranging from 0.88 to 2.01 indicating a tendency toward a higher item agreement to positive effects of the screening. This pattern is in line with fewer floor effects for the positive items in comparison to the negative items, so that substantial floor effects were found for item 13, 14, 15, 21, and 22, moderate effects were found for items 18, 19 and 20 and small floor effects were found for items 16 and 17. Substantial ceiling effects were only found for item 16, apart from that, the ceiling effects for the positive PCQ scales can be considered as small to moderate in general. Regarding skewness and kurtosis, the positive items show values closer to zero than the negative items. However, most of them exceed the cut-off values ± 1.96, indicating no normal distribution of the data. One should notice that all positive PCQ items show negative signs for kurtosis.

### Internal consistency

For this section and the subsequent analyzes, we applied missing value imputation as stated above. Furthermore, we recoded the positive items to compute the subscale indices so that higher scale values indicate less positive impact. In general, we found acceptable McDonald’s Omega higher than 0.7, except for the overall physical subscale, the overall social subscale and the social positive subscale (see Table 2) (Moosbrugger and Kelava, 2020). For the physical subscale, higher McDonald’s Omega values can be found if they were treated separately (positive and negative subscale). The social subscale shows major problems, since the coefficient could not be computed due to zero to negative covariances and the social positive subscale has only two items. For the emotional dimension we found a good Omega for the overall scale (0.80) with a better Omega for the negative scale (0.94) and a slightly worse Omega for the positive emotional scale (0.79). This pattern is also reflected in the mean inter-item correlation. In general, the overall subscales show lower values than if treated separately. Regarding the total PCQ scales (positive, negative, total), we found excellent McDonald’s Omega values of 0.86 and higher indicating good consistency. For the negative subscales and the total negative PCQ scale, we found strong deviation between the scale mean and the median indicating that the majority of our sample is not affected strongly in general, but bias by few extreme values occurs.

**TABLE 2:** Internal consistency: German version of the Psychological Consequences of Screening Questionnaire (PCQ).

Scale and items	N	Mean (SD)	Theoretical range	Observed range	Items	Item-total correlation	Mean inter-item correlation	McDonald’s Omega	25th percentile	Median	75th percentile
Physical (overall)	443	8.3 (4.3)	0–21	0–21	7	0.39–0.52	0.29	0.55	5	8	10
Includes items 01, 02, 10, 11, 16, 17, 21											
Physical (negative)	443	2.4 (3.1)	0–12	0–12	4	0.60–0.78	0.61	0.86	0	0	4
Includes items 01, 02, 10, 11											
Physical (positive)	443	5.9 (2.8)	0–9	0–9	3	0.63–0.81	0.67	0.87	0	6	8
Includes items 16, 17, 21											
Emotional (overall)	443	10.5 (6.4)	0–30	0–30	10	0.31–0.70	0.36	0.80	5	9	15
Includes items 03, 04, 05, 06, 12, 13, 14, 18, 19, 22											
Emotional (negative)	443	3.4 (4.4)	0–15	0–15	5	0.77–0.89	0.78	0.94	0	1	6
Includes items 03, 04, 05, 06, 12											
Emotional (positive)	443	7.2 (3.8)	0–15	0–15	5	0.45–0.66	0.44	0.79	4	7	9
Includes items 13, 14, 18, 19, 22											
Social (overall)	443	5.2 (2.9)	0–15	0–15	5	0.28–0.44	0.23	*	3	5	6
Includes items 07, 08, 09, 15, 20											
Social (negative)	443	1.3 (2.1)	0–9	0–9	3	0.58–0.73	0.57	0.82	0	0	2
Includes items 07, 08, 09											
Social (positive)	443	3.8 (2.0)	0–6	0–6	2	0.66–0.66	0.66	**	2	4	5
Includes items 15, 20											
Total PCQ (negative)	443	7.1 (8.6)	0–36	0–36	12	0.57–0.86	0.61	0.95	0	2	13
Includes items 01, 02, 03, 04, 05, 06, 07, 08, 09, 10, 11, 12											
Total PCQ (positive)	443	16.91 (7.5)	0–30	0–30	10	0.30–0.79	0.46	0.90	11	17	23
Includes items 13, 14, 15, 16, 17, 18, 20, 21, 22											
Total PCQ	443	23.0 (12.2)	0–66	0–66	22	0.33–0.66	0.30	0.86	15	24	30
Includes all 22 items											

*McDonald’s Omega could not be estimated because of item covariances of 0 or negative.

**McDonald’s Omega could not be estimated due to item number < 3.

### Scale structure

Table 3 shows the model fit statistics of our confirmatory factor analyses. First, we calculated strict 3-factor solutions not allowing covariances between the three dimensions. All model fit statistics show bad values for all three models indicating a misspecification of each of the models. Second, we estimated one-factor solutions for each subscale and the overall scale. In general, the fit statistics are better for each model, however, the overall scale model has to be considered as misspecified. For the negative and the positive subscales, we reached a strong decrease of Chi^2^, which still has a significant *p*-value indicating non-optimal fit. Though TLI and CFI reached the pre-defined cut-off-values for both the negative and the positive model, RMSEA and SRMR did not meet the cut-off values for the positive subscale model.

**TABLE 3:** Global Fit Indices of Confirmatory Factor Analysis: German version of the Psychological Consequences of Screening Questionnaire (PCQ) - weighted least square mean and variance adjusted estimators (WLSMV).

	N	χ ^2^ (robust)	df	X^2^/df	P	Scaling correction factor	TLI (robust)	CFI (robust)	RMSEA (robust)	RMSEA 90% CI	*P*-value RMSEA < = 0.05	SRMR (robust)
strict 3-factor CFA model (negative)	443	9991.34	54	185.02	0.000	2.85	0.56	0.64	0.65	0.64–0.66	0.000	0.56
1-factor CFA model (negative)	443	408.34	54	7.56	0.000	0.78	0.99	0.99	0.12	0.11–0.13	0.000	0.05
higher order CFA model (negative)	443	220.26	51	4.32	0.000	0.54	0.99	0.99	0.09	0.08–0.10	0.000	0.04
Modified higher order CFA model (negative)^a^	443	155.64	50	3.11	0.000	0.49	0.99	0.99	0.07	0.06–0.08	0.005	0.03
strict 3-factor CFA model (positive)	443	13985.72	35	399.59	0.000	NA	0.000	-0.899	0.95	0.94–0.96	0.000	0.42
1-factor CFA model (positive)	443	729.72	35	20.84	0.000	0.66	0.91	0.93	0.21	0.20–0.23	0.000	0.11
higher order CFA model (positive)	443	587.36	32	18.36	0.000	0.60	0.92	0.94	0.20	0.18–0.21	0.000	0.09
Modified higher order CFA model (positive)^b^	443	240.09	30	8.00	0.000	0.50	0.97	0.98	0.13	0.11–0.14	0.000	0.06
strict 3-factor CFA model (overall)	443	14492.12	209	69.34	0.000	2.98	0.55	0.51	0.39	0.39–0.40	0.000	0.43
1-factor CFA model (overall)	443	4946.06	209	23.67	0.000	2.22	0.84	0.85	0.23	0.22–0.23	0.000	0.37
higher order CFA model (overall)	443	4704.32	206	22.84	0.000	2.22	0.84	0.86	0.22	0.22–0.23	0.000	0.37
Modified higher order CFA model (overall)^c^	−	−	−	−	−	−	−	−	−	−	−	−

^a^As modification indices suggested that model fit would be improved if correlated error terms were included, we added one error term correlations that could improve the model the most (e10 and e11).

^b^As modification indices suggested that model fit would be improved if correlated error terms were included, we added two error term correlations that could improve the model the most (e13 and 14, e16 and 17).

^c^As many modification indices suggested a broad improvement of the model, we decided not to further modify a model that is apparently misspecified.

We estimated the unstandardized covariance between the assumed three factors for all three setups: For the negative PCQ dimensions, we found strong covariance between the physical and the emotional dimension (0.68), between the physical and social dimension (0.64) and even stronger between the emotional and social dimension (0.73). For the positive items, the covariances between the latent constructs were smaller between the emotional and physical domain (0.35) and between the emotional and social domain (0.32). The physical and social domain showed high covariance (0.76). For the overall model, we also found moderate to high covariances between the physical and emotional scale (0.55) as well as between the physical and the social scale (0.56) and between the emotional and social scale (0.63). Altogether, these findings support the use of a higher-order CFA allowing covariances between the three factors.

Thus, we modeled higher order CFA in a third step with three assumed latent constructs on level 1 and one overall construct on level 2. The model fit statistics increased slightly both for the positive and the negative subscales, indicating that the higher order CFA solution is fitting better than the one-factor solution. For the overall scale model, the performance remains weak and insufficient.

**FIGURE 1:**
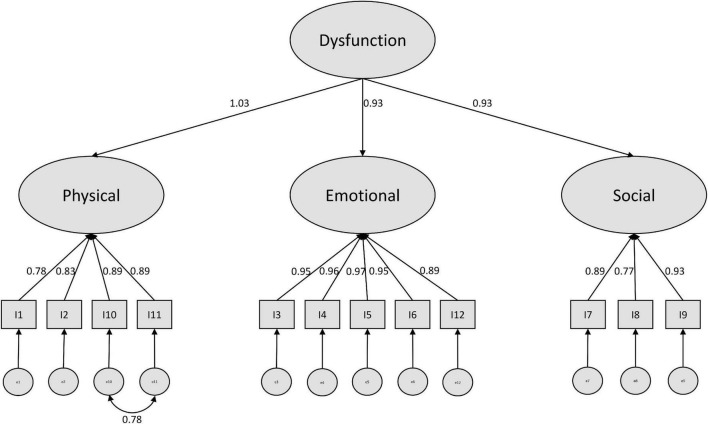
Modified higher order CFA model with standardized estimates (negative subscale).

In a last step, we modified our models based on suggestions by the modification indices (see Supplementary Material). For the negative model, a covariance of the error terms of item 10 and 11 (physical subscale) was recommended. We consider this as plausible, since both items refer to difficulties maintaining daily life activities, while the other two items of this construct refer to nutritional and sleeping impairments. Further modification indices suggest cross-loadings of item 12 with the physical and the social scale. However, since we did not find a clear rationale for this, we stopped modification here and ended up with a well performing modified model (see Figure 1).

**FIGURE 2:**
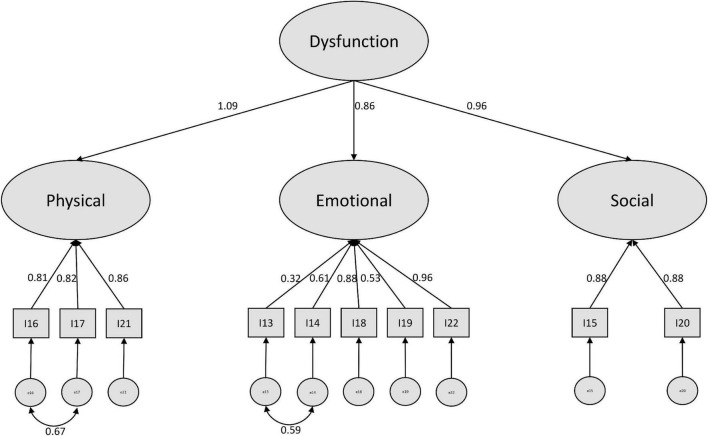
Modified higher order CFA model with standardized estimates (positive subscale).

For the positive subscale, we found a higher amount of modification suggestions. The highest reduction in Chi^2^ was suggested by implementing a covariance between the error terms of items 13 and 14. Here the phrasing of the items might play a role. Both items were formulated beginning with “I feel” in German. This is also true for item 22, however this refers to an overall expression, while item 13 and 14 are more specific. After including this covariance, the covariance of item 16 and 17 was suggested. Since those two items also refer to daily life activities (as in the negative items 10 and 11), we included this variance as well. After modification, we ended up with a sufficiently performing model (see Figure 2).

For the overall CFA model, we decided not to implement further modifications. The model seems to be strongly misspecified, so that many modifications would be necessary to reach acceptable model fit. However, this would pose the risk of overfitting a model and constructing a statistical artifact that might be far away from reality.

Since we could reach a better model fit for both the negative and the positive models, when implementing a higher order CFA, we consider that as a prime example of the bio-psycho-social model (Schotte et al., 2006) which indicates the difficulty of separating the three dimensions physical, social and emotional from each other.

### Convergent validity

As exposed in Table 4, we have to reject our hypotheses partially. Only the emotional subscale shows a strong correlation (*r* = 0.53) with the STAI-Y-6. The physical and social overall subscales show moderate positive correlations with the STAI-Y-6 ranging from 0.40 to 0.42. However, the total PCQ scale fits the hypothesis so that a strong correlation of 0.52 can be found. As we assumed, all positive subscales correlate less strongly with the STAI-Y-6 than the negative subscales do. Since we also found evidence for the assumption that the emotional subscale is stronger related to the STAI-Y-6 than the other subscales are, we overall can assume acceptable convergent validity of the PCQ.

**TABLE 4:** Concurrent validity: German version of the Psychological Consequences of Screening Questionnaire (PCQ).

	Emotional (neg)	Emotional (pos)	Emotional (overall)	Physical (neg)	Physical (pos)	Physical (overall)	Social (neg)	Social (pos)	Social (overall)	PCQ (neg)	PCQ (pos)	Total PCQ
STAI-Y Short version	0.498***	0.317***	0.528***	0.416***	0.178**	0.424***	0.393***	0.153***	0.402***	0.485***	0.265***	0.517***

Pearson’s correlations, *p < 0.05, **p < 0.01, ***p < 0.001. Interpretation guide: high STAI values indicate higher anxiety. Higher negative PCQ values (and on each subscale) indicate higher dysfunction. Higher positive PCQ values (and on each subscale) indicate less positive effect. N = 372.

## Discussion

Overall, our study results report a successful adaptation of the German PCQ with good performance in terms of acceptability, internal consistency, scale structure and convergent validity. The non-response rates and non-unique answer rates were negligible. Our results were comparable to other adaptations of the PCQ (Brodersen and Thorsen, 2003; Brodersen, 2006; Rijnsburger et al., 2006) and demonstrate that the PCQ is not only useful for the setting of cancer diseases.

However, as in other validation studies, we found substantial floor effects, especially for the negative PCQ items (Rijnsburger et al., 2006). Floor effects pose a potential risk in terms of accuracy of a scale since one has to assume that some kind of variation is drawn together in the lowest category. In general, we would suggest further differentiating the response categories, but this would also have as consequence that homogeneity of measurement across studies and countries would suffer. Since the lowest category is “not at all” one can assume that the PCQ produces floor effects if the population simply does not experience the eligible dysfunction. Here, it seems that the overall psychosocial impact of the screening is quite low.

Another limitation of the PCQ is its content validity. Brodersen and Thorsen (2008) found in a similar study translating and adapting the PCQ into Danish, that the original items do not cover all psychosocial aspects of screening. This is especially the case for negative consequences of abnormal screening. Therefore, an enhanced version of the PCQ containing 33 items was suggested. However, they extended questions covering areas that are exclusively relevant to the context of breast cancer screening. For future research we recommend to follow the approach of Brodersen and Thorsen (2008) using focus groups to check for potential uncovered fields of the PCQ in the context of liver diseases. Using this method could also reveal insights in whether diagnosis of early cirrhosis is equivalent to cancer screening regarding the patient reported outcome measures, which was an assumption we need to made for this study.

Reliability analyses suggest not summing up the physical, social and emotional subscales for positive and negative items. Moreover, the separation of the two scales should be applied as intended by Cockburn et al. and confirmed by Ong et al. (1997) into a negative and a positive subscale (Cockburn et al., 1992). This was also well demonstrated in our CFA, since the overall model was extremely misspecified and the separate treatment of positive and negative items reached clearly better fit. Regarding the discussion whether a three-factor or a one-factor solution is more favorable, our structural equation modeling approach suggested a compromise by treating the scales as higher order factors. Our analyses showed that a three-factor solution not allowing covariances between the three factors (which were found to be strong in our study, as well as proven in earlier psychometric tests of the PCQ (Cooper and Aucote, 2009) leads to weak performing CFA. If necessary, the one-factor solution is a better choice, however, we recommend to model the higher order factor structure as we presented in this work.

Because of the cut-off value of 80%, which we chose as a criterion for too incomplete scales for analysis, we had to exclude 29 cases only because of the positive subscales and four cases only because of the negative items. In line with a generally higher non-response rate of the positive items, we assume that here fatigue effects might have occurred. The positive items were placed as a block after the negative items in our questionnaire at the end of the page. Since our study could not apply randomization of items, we recommend further testing of the German version of the PCQ including tests for order effects.

Another limitation of our study design was that we could not contact the patients in a specific period after their screening so that some patients have longer periods between the screening experience and responding to the questionnaire than other ones. An individual contacting procedure (e.g., 4 weeks after each test) would improve the comparability of the measurement, but was not realistic to implement in this study. We recommend further testing the German PCQ in a controlled setting, which could also offer the possibility to get insights into retest reliability and the sensitivity of measuring changes over time. This could shed light on the progress of potential burden after a screening experience.

The general low response rate to our study limits representativeness of our results. After the delivery of the questionnaire, we received some phone calls of patients who were not aware of their inclusion into this study. Cognitive impairment and lingual problems also were named as reasons for non-response to our questionnaires.

Due to the weak performance of the modified CFA model for the total PCQ and because of the shared variance of items 9, 10, 11 and the emotional subscale, a pattern that occurs only in the total PCQ model, we recommend not to use the total PCQ score and instead computation of the negative and the positive PCQ separately.

Since the negative PCQ subscale and the total PCQ correlated at least moderately with the STAI-Y-6, we consider the convergent validity as acceptable. However, more insights into other aspects of validity would help to further evaluate the performance of the PCQ. In other validation studies, the Impact of Event Scale (IES) and the Hospital Anxiety and Depression Scale (HADS) were used for validation. In our project, the IES seemed not to be adequate since most of our participants were screened negatively. Thus, the considered event “liver cirrhosis” is not as present as it is in the context of the breast cancer screening study of Rijnsburger et al. (2006). The HADS was also not considered to be useful in our context since it contains a longer list of items than the short form of the STAI. However, for the future investigation of the PCQ we recommend also considering the HADS or IES to enhance international comparability of the German version of the PCQ.

## Conclusion

Overall, our study results report a successful adaptation of the German PCQ with good performance in terms of acceptability, internal consistency, scale structure, and convergent validity. We could demonstrate that the German version of the PCQ is a useful and well-performing measurement for both negative and positive screening consequences, even in a non-cancer setting. However, future studies need to address content validity of the PCQ in the context of liver screening.

## Data availability statement

The raw data supporting the conclusions of this article can be requested from the corresponding author.

## Ethics statement

The studies involving human participants were reviewed and approved by Ethics Committees of Rhineland-Palatinate and Saarland, Germany. The patients/participants provided their written informed consent to participate in this study.

## Author contributions

UF wrote the draft of the manuscript and was responsible for survey design, data collection and analysis. AM and EF-G reviewed the manuscript and revised it critically. EF-G was project leader and mentored the development of the questionnaire used in the project. UF and EF-G translated the questionnaire into German and reconciled their versions. All authors contributed to the article and approved the submitted version.

## Footnotes

1 Rijnsburger et al. used the Hospital Anxiety and Depression Scale as well as the Impact of Event Scale for testing construct validity. Due to the nature of the SEAL project, we considered the STAI-Y-6 as more suitable in our context.

## Abbreviations

PCQ: psychological consequences questionnaire
SEAL: structured early detection of asymptomatic cirrhosis of the liver in Rhineland-Palatinate and Saarland
APRI: aspartate aminotransferase to platelet ratio
PRO: patient-reported outcome
CFA: confirmatory factor analysis
WLSMV: weighted least square mean and variance adjusted
DWLS: diagonally weighted least squares
CFI: comparative fit index
TLI: Tucker-Lewis index
RMSEA: root mean square error of approximation
SRMR: standardized root mean square residual
STAI-Y-6: Spielberger state-trait anxiety inventory (Short Form)
IES: Impact of event scale
HADS: Hospital anxiety and depression scale.
